# Abnormal expression and methylation of *PRR34‐AS1* are associated with adverse outcomes in acute myeloid leukemia

**DOI:** 10.1002/cam4.4085

**Published:** 2021-07-05

**Authors:** Fang‐yu Nan, Yu Gu, Zi‐jun Xu, Guo‐kang Sun, Jing‐dong Zhou, Ting‐juan Zhang, Ji‐chun Ma, Jia‐yan Leng, Jiang Lin, Jun Qian

**Affiliations:** ^1^ Department of Hematology Affiliated People’s Hospital of Jiangsu University Zhenjiang Jiangsu People’s Republic of China; ^2^ Laboratory Center Affiliated People’s Hospital of Jiangsu University Zhenjiang Jiangsu People’s Republic of China; ^3^ Zhenjiang Clinical Research Center of Hematology Zhenjiang Jiangsu People’s Republic of China; ^4^ West China School of Public Health and China Fourth Hospital Sichuan University Chengdu Sichuan People’s Republic of China

**Keywords:** acute myeloid leukemia, DNA methylation, expression, lncRNAs, prognosis, *PRR34‐AS1*

## Abstract

It was previously reported that *PRR34*‐*AS1* was overexpressed in some solid tumors. *PRR34*‐*AS1* promoter was shown to have a differential methylation region (DMR), and was hypomethylated in acute myeloid leukemia (AML). Therefore, the present study used real‐time quantitative PCR (RQ‐PCR) to explore the expression characteristics of *PRR34*‐*AS1* in AML. In addition, the correlation between the expression of *PRR34*‐*AS1* and clinical prognosis of AML was determined. The findings of this study indicated that high *PRR34*‐*AS1* expression was bound up with shorter overall survival (OS) in AML patients (*p* = 0.002). Moreover, patients with high expression of *PRR34*‐*AS1* had significantly lower complete remission (CR) rate compared with those with low expression of *PRR34*‐*AS1* after induction chemotherapy. Furthermore, multivariate analysis confirmed that *PRR34*‐*AS1* expression was an independent factor affecting CR in whole‐AML, non‐APL‐AML, and CN‐AML patients (*p* = 0.032, 0.039, and 0.036, respectively). Methylation‐specific PCR (MSP) and bisulfite sequencing PCR (BSP) were used to explore the methylation status of *PRR34*‐*AS1*. *PRR34*‐*AS1* promoter showed a pattern of hypomethylation in AML patients compared with normal controls (*p* = 0.122). Notably, of whole‐AML and non‐APL‐AML patients, *PRR34*‐*AS1* hypomethylated patients presented a significantly shorter OS than those with a hypermethylated *PRR34*‐*AS1* (*p* = 0.010 and 0.037, respectively). Multivariate analysis confirmed that the hypomethylation of *PRR34*‐*AS1* served as an independent prognostic indicator in both whole‐cohort AML and non‐APL‐AML categories (*p* = 0.057 and 0.018, respectively). In summary, the findings of this study showed that abnormalities in *PRR34*‐*AS1* are associated with poor prognosis in AML. Therefore, monitoring this index may be important in the prognosis of AML and can provide information on effective chemotherapy against the disease.

## Graphical abstract

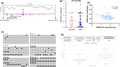

## 1 INTRODUCTION

Acute myeloid leukemia (AML) presents with features of the accumulation of myeloid leukemia cells in bone marrow (BM), blood, and other tissues, The disease mainly results in poorly differentiated erythrocytes, platelets, and white blood cells (WBC) in the BM.^1^ In addition, AML can occur at all ages although the incidence rate is highest in the elderly (>60 years).^2^ Cytogenetic analysis has been conventionally used to study the molecular pathogenesis of leukemia for more than 50 years since the 1960s.^3^ In addition, cytogenetic findings are reported to be important diagnostic and prognostic markers.^4^ However, almost half of AML patients have normal karyotypes. Advances in targeted sequencing technology have led to the identification of some genetic mutations such as *FLT3*, *NPM1*, *KIT*, *CEBPA*, and *TET2* in AML.^5^ Most previous studies mainly focused on protein‐coding genes to explore the molecular genetic changes and identify the prognostic markers of AML.^6^ However, the molecular mechanisms underlying the occurrence and development of AML have not been fully elucidated due to the high degree of heterogeneity in the disease.^7^, ^8^, ^9^ Therefore, exploring the pathogenesis of AML is important for the development of better treatment strategies and for improving the prognosis of patients.

Abnormal regulation of long non‐coding RNAs (lncRNAs) was involved in each stage of tumor occurrence, development, and migration.^10^ Moreover, genome‐wide association studies (GWAS) on tumor samples showed that numerous lncRNAs are associated with various types of cancer.^11^ LncRNAs play a significant role in promoting or inhibiting the development of AML.^6^ Carcinogenic lncRNAs include *H19*,^12^, ^13^ *HOTAIR*,^14^, ^15^, ^16^ and *PVT*‐*1* ^17^, ^18^ whereas tumor‐suppressing lncRNAs include *NEAT1*,^19^, ^20^ *IRAIN*,^21^ and *MEG3*.^22^, ^23^, ^24^

*PRR34* antisense RNA 1 (*PRR34*‐*AS1)* was shown to be upregulated in hepatocellular carcinoma and pediatric medulloblastoma.^25^, ^26^ In addition, cholangiocarcinoma patients with high expression of *PRR34*‐*AS1* were reported to have a shorter disease‐free survival (DFS). Analysis of possible mechanism showed that *PRR34*‐*AS1* acted through the JAK‐signal transducer and activated the transcription of factors in the JAK‐STAT pathway.^27^ Furthermore, *PRR34*‐*AS1* was shown to exert its effects through the JAK‐STAT signaling pathway after total knee arthroplasty (TKA) ischemia/reperfusion (I/R) injury.^28^ The differential methylation region (DMR) in the *PRR34*‐*AS1* promoter was reported to be hypomethylated in AML.^29^ However, the direct role and clinical significance of *PRR34*‐*AS1* expression in AML have not been fully elucidated. Moreover, *PRR34*‐*AS1* promoter methylation status and its clinical correlation with AML should be explored. Therefore, the present study sought to explore the expression and methylation characteristics of *PRR34*‐*AS1* and their clinical significance in AML.

## 2 MATERIALS AND METHODS

### 2.1 Patients’ samples

A total of 84 newly diagnosed AML adult patients and 29 healthy controls from our hospital were enrolled in this study. Participants provided written informed consent prior to the study and ethical approval was obtained from the hospital's ethical committee. Diagnosis and classification of cases in the study were performed based on the 2016 World Health Organization (WHO) criteria. The treatment protocol is listed in Table S1.

### 2.2 Real‐time quantitative PCR

Bone marrow mononuclear cells (BMMNCs) were obtained using density gradient centrifugation. Total RNA was extracted from BMMCs. Reverse transcription of RNA was performed to generate cDNA,^30^ following a protocol described previously.^31^ Real‐time quantitative PCR (RQ‐PCR) was used to determine the expression levels of *PRR34*‐*AS1* in the BMMNCs. The following upstream and downstream primer sequences were used to determine the expression levels of *PRR34*‐*AS1*; 5’‐GAGGCCATCTTTGGAAAGTAAA‐3’ and 5’‐AACGATGTGAGCCGAGCA‐3’, respectively. RQ‐PCR was conducted using a 20 µl reaction volume containing 20 ng of cDNA, 0.8 µM of primers, 6 µl of H_2_O, 10 µM of SYBR Premix TB Green (Takara), and 0.4 µM of ROX Reference Dye II (Takara). RQ‐PCR reaction conditions were as follows; 95°C for 30 s followed by 40 cycles of 95°C for 5 s, 61°C for 32 s, finally 95°C for 15 s, 60°C for 60 s, 95°C for 15 s, and 60°C for 15 s. Each test included a negative control and positive control, and false positives and false negatives were excluded, respectively. *ABL1* was used as the internal reference gene, and *PRR34*‐*AS1* transcript levels in various samples were determined using the 2^−ΔΔCT^ method.

### 2.3 DNA isolation, bisulfite modification, and methylation‐specific PCR

Genomic DNA was extracted from samples obtained from AML patients, AML culture cells, and normal controls using the Genomic DNA Purification Kit (Gentra). Genomic DNA was modified using the CpGenome DNA modification kit (Chemicon). Methylation‐specific PCR (MSP) was used to explore the methylation status of the *PRR34*‐*AS1* promoter. Forward and reverse primer sequences used for methylated *PRR34*‐*AS1* (M‐*PRR34*‐*AS1*) were 5’GGAAATGTTTAGGTCGAGGC‐3’ and 5’‐CACACATCAAAACGAAAACG‐3’, respectively. Upstream and downstream primer sequences for unmethylated *PRR34*‐*AS1* (U‐*PRR34*‐*AS1*) were; 5’‐TATGGAAATGTTTAGGTTGAGGT‐3’ and 5’‐CACACACATCAAAACAAAAACAA‐3’, respectively. The reaction conditions were as follows; 95°C for 30 s followed by 40 cycles of 95°C for 5 s, 61°C for 32 s, 72°C for 30 s, and 78°C for 32 s. *PRR34*‐*AS1* methylation levels were then calculated using the $2^{‐\Delta \Delta \text{CT}}$ method.

### 2.4 Bisulfite sequencing PCR

Bisulfite sequencing PCR (BSP) was used to explore the density of methylation in *PRR34*‐*AS1* and evaluate the accuracy of MSP. Notably, investigating differential methylation through BSP is a key step in the analysis of epigenetic data.^32^ Upstream and downstream primer sequences for BSP were; 5’‐TTGGTATGGGAGGAGTTAAGTT‐3’ and 5’‐AAATCCCAACAACCATATACAA‐3’, respectively. The reaction conditions of BSP were pre‐denaturation (98°C for 10 s), denaturation (98°C for 10 s), annealing (61°C for 30 s), elongation (72°C for 30 s), and enzyme inactivation (72°C for 7 min). The number of cycles was set at 40. After purification and recovery, the recombinant vector was constructed using the pMD^®^19‐T vector (Takara), then transfected into DH5α competent cells (Vazyme Biotech Co.) for cloning. Sequences of six independent clones from each specimen were verified (BGI Gene Technology Co., Ltd.).

### 2.5 Gene mutation detection

LightScanner software was used to design specific primers for gene hot spots. High‐resolution melt analysis (HRMA) was used to examine mutations in *N /K‐RAS*, *IDH1*/*2*, *DNMT3A*, *U2AF1*, *NPM1*, and *C*‐*KIT*.^33^, ^34^, ^35^, ^36^ Mutation and mutation type were determined by observing the melting curve and Tm shift. Direct DNA sequencing was used to assess mutations in *CEBPA* and *FLT3*‐*ITD*,^37^ and to verify all positive specimens.

### 2.6 Bioinformatics analyses

*PRR34*‐*AS1* mRNA expression (RNA Seq V2 RSEM) and methylation (HM450) data were retrieved from a cohort of 200 AML patients in the Cancer Genome Atlas (TCGA)^5^ and downloaded through the cBioPortal tool (http://www.cbioportal.org).^38^, ^39^ GenomicScape (http://genomicscape.com/) webserver was used for GEP analysis to further verify the relationship between the expression levels of *PRR34*‐*AS1* and prognosis of AML. Differential methylation analysis was accomplished by the Disease Meth version 2.0 tool (http://www.bio‐bigdata.com/diseasemath/analysis.html).

### 2.7 Statistical analysis

Data analysis was conducted using SPSS version 22.0 software (SPSS) and GraphPad Prism 8.0. Mann–Whitney U test was used to perform comparisons between continuous variables. Comparison between the two groups of categorical variables was conducted using the Pearson's chi‐square analysis test or the Fisher exact test. Receiver operating characteristic (ROC) curve and area under the ROC curve (AUC) were used to explore differences in levels of *PRR34*‐*AS1* methylation between AML patients and controls. Survival analysis was conducted using Kaplan–Meier survival estimates. Cox regression analysis was performed to evaluate the effect of expression and methylation of *PRR34*‐*AS1* on the clinical outcomes of AML patients. Finally, the Spearman's rank correlation analysis was used to examine the correlation between the two groups of variables (expression and methylation of *PRR34*‐*AS1* in AML patients). A *p* value <0.05 was considered to be statistically significant (bilateral).

## 3 RESULTS

### 3.1 Associations between the expression of *PRR34*‐*AS1* and clinical as well as laboratory characteristics in AML patients

Expression levels of *PRR34*‐*AS1* in AML patients were determined using RQ‐PCR. Analysis showed that the transcript levels of *PRR34*‐*AS1* ranged from 0.000 to 20.339 (median 0.613) in 83 newly diagnosed AML patients. To explore the clinical characteristics of *PRR34*‐*AS1*, the cohort was group into high and low‐expression groups using the median value of the level of expression as the cutoff. The findings showed that high expression of *PRR34*‐*AS1* was associated with higher levels of WBC (*p* = 0.041), platelets (*p* = 0.004), and older age (*p* < 0.001, Table 1). Moreover, there was a significant difference in the classification of karyotypes between the groups (*p* = 0.043). In addition, the frequency of favorable karyotypes in the high *PRR34*‐*AS1* expression group was relatively lower compared with the level for the low‐expression group; however, the difference was not significant (14% vs. 32%, *p* = 0.059; Table 1).

**TABLE 1:** Comparison of clinical manifestations and laboratory features between AML patients with low and high *PRR34*‐*AS1* expression

Patient's parameters	Low (*n* = 41)	High (*n* = 42)	*p* value
Sex, male/female	20/21	28/14	0.101
Median hemoglobin, g/L (range)	77 (34–141)	80 (49–131)	0.600
Median age, years (range)	48 (22–84)	60 (29–85)	<0.001
Median WBC, ×10^9^/L (range)	6.9 (0.3–140.2)	27.65 (0.8–528.0)	0.041
Median platelets, ×10^9^/L (range)	25 (3–144)	49 (9–415)	0.004
BM blasts, % (range)	42.5 (3–91.0)	37 (6.5–97.5)	0.212
FAB subtypes			0.028
M0	0 (0%)	1 (2.4%)	
M1	1 (2.4%)	1 (2.4%)	
M2	15 (36.6%)	15 (35.7%)	
M3	11 (26.8%)	3 (7.1%)	
M4	5 (12.2%)	9 (21.4%)	
M5	1 (2.4%)	7 (16.7%)	
M6	0 (0%)	1 (2.4%)	
Karyotype classification			0.043
Favorable	13 (31.7%)	6 (14.3%)	
Intermediate	24 (58.5%)	32 (76.2%)	
Poor	4 (9.8%)	1 (2.4%)	
No data	0 (0%)	3 (7.1%)	
Karyotype			0.093
Normal	20 (48.8%)	24 (57.1%)	
t(8;21)	3 (7.3%)	2 (4.8%)	
t(15;17)	10 (24.4%)	3 (7.1%)	
t(9;22)	0 (0%)	1 (2.4%)	
+8	0 (0%)	2 (4.8%)	
−7/7q−	1 (2.4%)	0 (0%)	
complex	3 (7.3%)	1 (2.4%)	
others	4 (9.8%)	6 (14.3%)	
No data	0 (0%)	3 (7.1%)	
Gene mutation			
*CEBPA* (+/−)	3/32	4/22	0.689
*NPM1* (+/−)	4/28	3/23	>0.999
*FLT3*‐ITD (+/−)	3/29	5/21	0.446
*C*‐*KIT* (+/−)	2/30	0/26	0.497
*N*/*K*‐*RAS* (+/−)	0/24	2/21	0.234
*IDH1*/*2* (+/−)	0/32	1/25	0.448
*DNMT3A* (+/−)	2/30	2/24	>0.999
*U2AF1* (+/−)	0/32	1/25	0.448
CR (+/−)	24/9	13/22	0.004

Abbreviations: BM, bone marrow; CR, complete remission; FAB, French–American–British; WBC, white blood cells.

### 3.2 Association between the expression of *PRR34*‐*AS1* and efficacy of chemotherapy in AML patients

**FIGURE 1:**
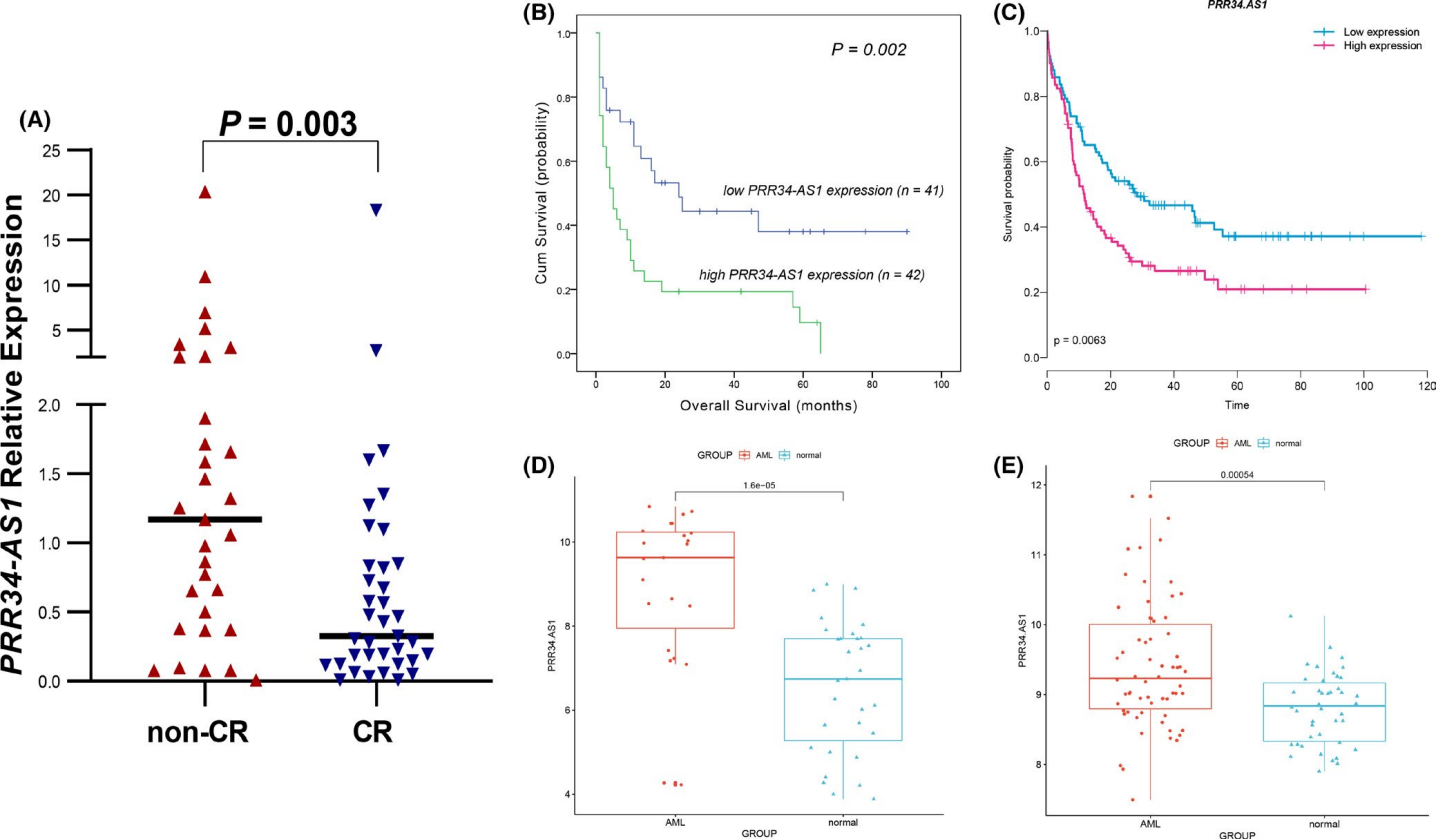
Predictive value of *PRR34*‐*AS1* expression on CR rate and OS in AML patients. (A) Expression of *PRR34*‐*AS1* in CR and non‐CR AML patients receiving induction therapy. (B) Overall survival (OS) of the whole cohort of AML patients. (C) Prognostic value of *PRR34*‐*AS1* expression for OS in whole AML patients from TCGA database (GSE68833, *n* = 183). (D, E) *PRR34*‐*AS1* expression level in AML patients and controls obtained from Gene Expression Omnibus (GEO)database. [D, GSE24006: AML = 23, Normal = 31; E, GSE63270: AML = 62, Normal = 42]

Analysis showed that patients with high expression of *PRR34*‐*AS1* had a lower CR rate compared with those with low expression of the gene [37.1% (13/22) vs. 72.7% (24/9), *p* = 0.004, Table 1]. In addition, expression levels of *PRR34*‐*AS1* were analyzed in patients with CR and those without CR after induction chemotherapy. Analysis showed that non‐CR patients had significantly higher levels of *PRR34*‐*AS1* compared with patients with CR (*p* = 0.03; Figure 1A). Further, differences in clinical characteristics of AML patients with and without CR were explored. The findings showed that patients in the non‐CR group were older, had high expression levels of *PRR34*‐*AS1* and higher levels of WBC and platelets compared with patients with CR (*p* = 0.010, 0.003, 0.009, and 0.004, respectively; Table 2). Moreover, the analysis showed a significant decrease in the frequency of favorable karyotypes in the non‐CR group compared with the CR group (9.7% vs. 40.5%, *p* = 0.005; Table 2).

**TABLE 2:** Comparison of clinical manifestations and laboratory features between CR and non‐CR in AML patients receiving induction therapy

Patient's parameters	Non‐CR (*n* = 31)	CR (*n* = 37)	*p* value
*PRR34*‐*AS1* expression	1.2 (0–20.3)	0.3 (0–18.3)	0.003
Sex, male/female	21/10	18/19	0.143
Median hemoglobin, g/L (range)	80 (49–138)	80 (34–131)	0.810
Median age, years (range)	60 (22–81)	48 (24–77)	0.010
Median WBC, ×109/L (range)	38.7 (0.9–185.4)	9.1 (0.8–528.0)	0.009
Median platelets, ×109/L (range)	42 (9–415)	32 (3–192)	0.004
BM blasts, % (range)	37.75 (6.5–92)	37 (3.0–97.5)	0.143
FAB subtypes			0.038
M0	1 (3.2%)	0 (0%)	
M1	2 (6.5%)	0 (0%)	
M2	13 (41.9%)	15 (40.5%)	
M3	2 (6.5%)	11 (29.7%)	
M4	8 (25.8%)	6 (16.2%)	
M5	5 (16.1%)	2 (5.4%)	
M6	0 (0%)	1 (2.7%)	
Karyotype classification			0.005
Favorable	3 (9.7%)	15 (40.5%)	
Intermediate	22 (71%)	21 (56.8%)	
Poor	4 (12.9%)	0 (0%)	
No data	2 (6.5%)	1 (2.7%)	
Karyotype			0.016
Normal	18 (58.1%)	16 (43.2%)	
t (8;21)	0 (0%)	5 (13.5%)	
t (15;17)	2 (6.5%)	10 (27%)	
t (9;22)	1 (3.2%)	0 (0%)	
+8	1 (3.2%)	1 (2.7%)	
−7/7q‐	1 (3.2%)	0 (0%)	
complex	3 (9.7%)	0 (0%)	
others	3 (9.7%)	4 (10.8%)	
No data	2 (6.5%)	1 (2.7%)	
Gene mutation			
*CEBPA* (+/‐)	3/20	4/24	>0.999
*NPM1* (+/‐)	3/20	3/25	>0.999
*FLT3*‐*ITD* (+/‐)	4/19	3/25	0.687
*C*‐*KIT* (+/‐)	0/23	2/26	0.495
*N*/*K*‐*RAS* (+/‐)	2/18	0/25	0.192
*IDH1*/*2* (+/‐)	1/22	0/28	0.451
*DNMT3A* (+/‐)	2/21	2/26	>0.999
*U2AF1* (+/‐)	1/22	0/28	0.451

Abbreviations: BM, bone marrow; CR, complete remission; WBC, white blood cells.

Furthermore, logistics regression analysis further showed that *PRR34*‐*AS1* expression could serve as an independent factor affecting CR in patients with whole AML, non‐APL‐AML, and CN‐AML (Table 3).

**TABLE 3:** Univariate and multivariate analyses of variables for complete remission in whole‐cohort AML patients, non‐APL‐AML, and CN‐AML

Variables	whole‐AML (*n* = 68)				non‐APL‐AML (*n* = 56)				CN‐AML (*n* = 45)			
		Univariate analysis		Multivariate analysis		Univariate analysis		Multivariate analysis		Univariate analysis		Multivariate analysis	
		OR (95% CI)	*p* value	OR (95% CI)	*p* value	OR (95% CI)	*p* value	OR (95% CI)	*p* value	OR (95% CI)	*p* value	OR (95% CI)	*p* value
WBC		0.199 (0.069–0.575)	0.003	0.228 (0.072–0.721)	0.012	0.252 (0.079–0.800)	0.019	0.196 (0.054–0.713)	0.013	0.273 (0.067–1.102)	0.068	1.005 (0.996–1.013)	0.311
Age		0.294 (0.103–0.843)	0.023	0.941 (0.257–3.445)	0.926	0.441 (0.141–1.383)	0.160	1.455 (0.343–6.168)	0.611	0.417 (0.103–1.679)	0.218	—	—
*PRR34*‐*AS1* expression		0.222 (0.079–0.620)	0.004	0.282 (0.089–0.895)	0.032	0.272 (0.086–0.859)	0.026	0.253 (0.069–0.933)	0.039	0.210 (0.050–0.879)	0.033	0.210 (0.050–0.879)	0.033
Karyotype risk		0.268 (0.102–0.706)	0.008	0.351 (0.132–0.932)	0.036	0.356 (0.111–1.142)	0.083	0.332 (0.095–1.158)	0.084	—	—	—	—
*CEBPA* mutation		1.111 (0.222–5.560)	0.898	—	—	1.714 (0.329–8.943)	0.522	—	—	0.917 (0.110–7.666)	0.936	—	—
*NPM1* mutation		0.888 (0.145–4.401)	0.798	—	—	1.200 (0.210–6.842)	0.837	—	—	0.917 (0.110–7.666)	0.936	—	—
*FLT3*‐*ITD* mutation		0.570 (0.114–2.856)	0.494	—	—	0.531 (0.085–3.310)	0.498	—	—	0.556 (0.077–4.009)	0.560	—	—
*DNMT3A* mutation		0.808 (0.105–6.228)	0.838	—	—	1.187 (0.150–9.408)	0.871	—	—	2.000 (0.159–25.115)	0.591	—	—

Note

Variables including age (≤60 vs. <60 years), WBC (≥30 × 10^9^ vs.<30 × 10^9^/L), *PRR34*‐*AS1* expression (low vs. high), karyotype risk (favorable vs. intermediate vs. poor), and gene mutations (mutant vs. wild type).

Multivariate analysis includes variables with *p* < 0.200 in univariate analysis.

Abbreviations: AML, acute myeloid leukemia; CI, confidence interval; CN‐AML, cytogenetically normal AML; HR, hazard ratio; non‐APL‐AML, non‐acute promyelocytic leukemia‐AML; WBC, white blood cells.

### 3.3 Association between the expression of *PRR34*‐*AS1* and outcomes in AML patients

The median overall survival (OS) time for all AML patients was 10 months (range 1–90 months). Kaplan–Meier survival analysis showed that AML patients with high expression of *PRR34*‐*AS1* had a significantly shorter OS than those with low expression of *PRR34*‐*AS1* (*p* = 0.002; Figure 1B). To further explore the effect of *PRR34*‐*AS1* expression on OS in AML patients, data from Gene Expression Omnibus (GEO; accession number GSE68833) were analyzed using GenomicScape online tool and similar results were obtained (Figure 1C). Cox regression analysis showed that high expression of *PRR34*‐*AS1* was not an independent risk factor for OS in whole‐AML patients (Table 4).

**TABLE 4:** Univariate and multivariate analyses of prognostic factors for overall survival in whole‐AML patients

Variables	whole‐AML (n=83)			
	Univariate analysis		Multivariate analysis	
	HR (95% CI)	*p* value	HR (95% CI)	*p* value
WBC	2.514 (1.380–4.582)	0.003	2.218 (1.221–4.029)	0.009
Age	2.754 (1.494–5.075)	0.001	1.173 (0.545–2.528)	0.683
*PRR34*‐*AS1*expression	2.447 (1.313–4.559)	0.004	1.573 (0.821–3.017)	0.172
Karyotype risk	2.054 (1.401–3.011)	<0.001	2.070 (1.351–3.170)	<0.001
*CEBPA* mutation	1.123 (0.389–3.247)	0.830	—	—
*NPM1* mutation	1.522 (0.579–3.999)	0.394	—	—
*FLT3*‐ITD mutation	1.113 (0.426–2.912)	0.827	—	—
*c*‐*KIT* mutation	1.241 (0.167–9.202)	0.833	—	—
*DNMT3A* mutation	1.228 (0.371–4.070)	0.737	—	—

Note

Variables including age (≤60 vs. <60 years), WBC (≥30 × 10^9^ vs. <30 × 10^9^/L), *PRR34*‐*AS1* expression (low vs. high), karyotype risk (favorable vs. intermediate vs. poor), and gene mutations (mutant vs. wild type).

Multivariate analysis includes variables with *p* < 0.200 in univariate analysis.

Abbreviations: CI, confidence interval; HR, hazard ratio; WBC, white blood cells.

### 3.4 Association between the expression of *PRR34*‐*AS1* and methylation of its promoter in AML

**FIGURE 2:**
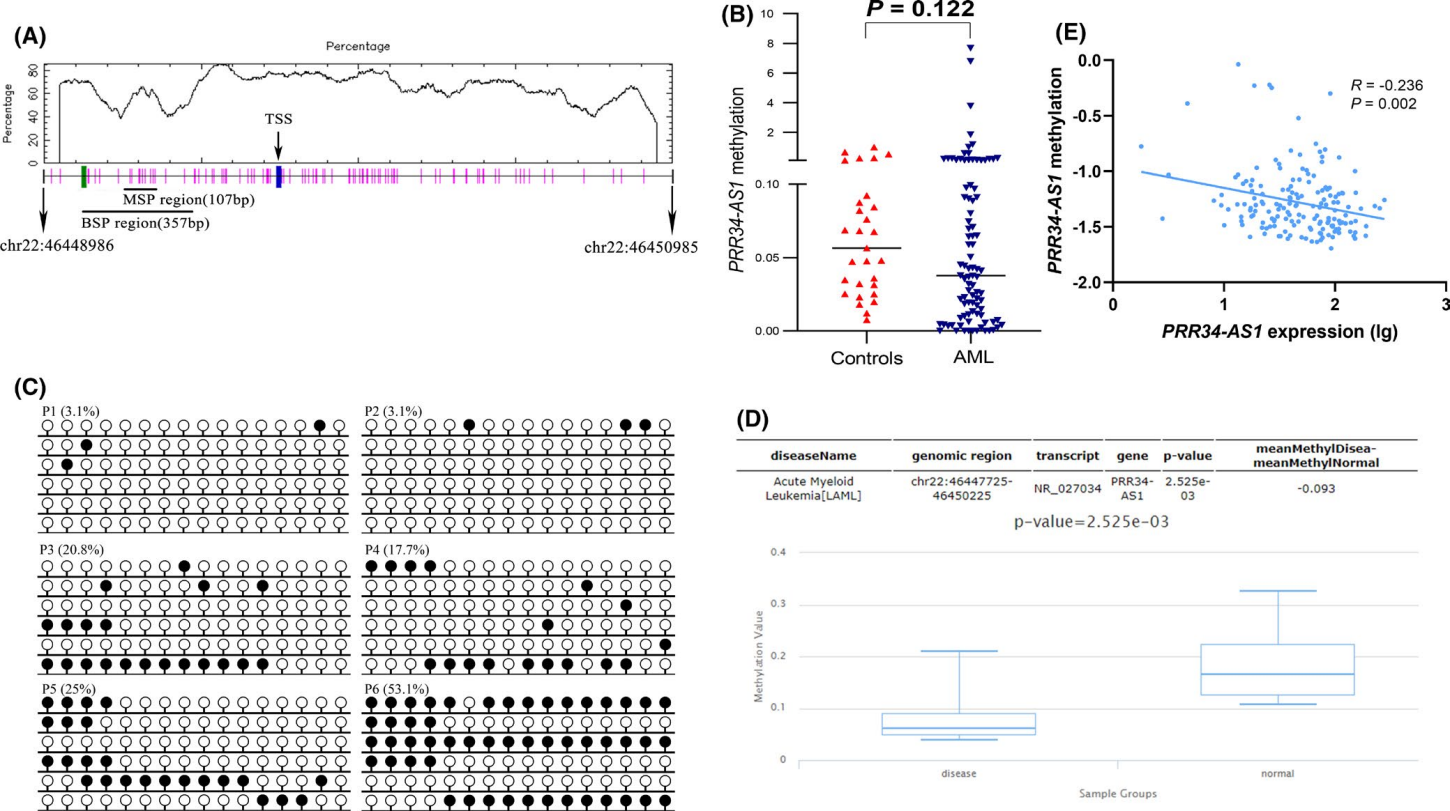
Validation of the methylation of *PRR34*‐*AS1* in AML. (A) A schematic diagram of the CpG island in the promoter region of *PRR34*‐*AS1*. Vertical bars indicate CpG dinucleotides. Short horizontal lines represent corresponding positions amplified by MSP and BSP primers. The figure was generated using cpgplot (http://emboss.bioinformatics.nl/cgi‐bin/emboss/cpgplot) and Methyl Primer Express V1.0 software. TSS: transcription start site; MSP: methylation‐specific PCR; BSP: bisulfite sequencing PCR. B: Methylation levels of *PRR34*‐*AS1* in the control group and AML patients were determined by MSP. (C) Methylation density of *PRR34*‐*AS1* detected by BSP. The white cycle indicates unmethylated CpG dinucleotides whereas the black cycle represents methylated CpG dinucleotides P1 and P2: unmethylated AML patients; P3 and P4: methylated AML patients; P4 and P5: controls. (D) Methylation status of *PRR34*‐*AS1* promoter (CpG island) was analyzed using Disease Meth version 2.0 tool (http://www.bio‐bigdata.com/diseasemeth/analyze.html). (E) Correlation analysis between *PRR34*‐*AS1* gene expression and its methylation in AML patients was analyzed using data from the TCGA database. Spearman test was used for correlation analysis

Analysis using GSE24006 and GSE63270 data sets showed high expression levels of *PRR34*‐*AS1* in both data sets (Figure 1D,E). The methylation status of the *PRR34*‐*AS1* promoter was determined to further explore whether changes in *PRR34*‐*AS1* methylation affected its expression. MSP and BSP primer sets were designed and verified on the CpG island of the *PRR34*‐*AS1* promoter region (Figure 2A). The methylation level of *PRR34*‐*AS1* was then determined through MSP in 84 AML patients and 29 normal controls. Analysis showed that *PRR34*‐*AS1* was hypomethylated in AML although there was no significant difference with normal control (*p* = 0.122; Figure 2B). Subsequently, two normal controls, two *PRR34*‐*AS1*‐hypermethylated AML patients, and two *PRR34*‐*AS1*‐unmethylated AML patients were randomly selected to verify the MSP results through BSP (Figure 2C). The unmethylated patients showed a completely unmethylated state in AML whereas hypermethylated patients and normal controls showed a higher density of methylation. Moreover, the degree of methylation in hypermethylated patients was lower compared with that in normal controls (Figure 2C). This implied that the results were consistent with MSP results. DiseaseMeth version 2.0 was used to determine the trend in the methylation of *PRR34*‐*AS1* promoter (CpG island) in AML. The results revealed that AML patients had significantly lower *PRR34*‐*AS1* methylation levels than the controls (Figure 2D). Furthermore, Spearman's rank test was used to analyze the correlation between the methylation and expression of *PRR34*‐*AS1* in AML patients using the TCGA data sets. The findings showed that there was a significant negative correlation between the methylation and expression of *PRR34*‐*AS1* (R = −0.236, *p* = 0.027, *n* = 168; Figure 2E). This finding implies that the aberrant methylation of *PRR34*‐*AS1* may be an important mechanism for regulating its expression in AML.

### 3.5 Association between *PRR34*‐*AS1* methylation and different clinical parameters in AML patients

**FIGURE 3:**
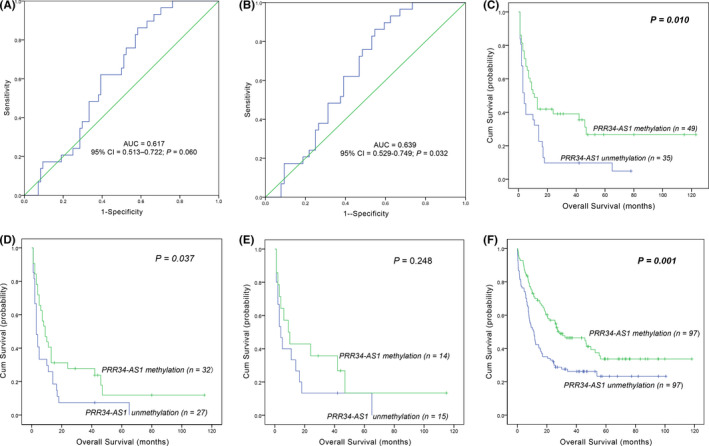
Effect of aberrant methylation of *PRR34*‐*AS1* on the prognosis of AML. (A, B) A ROC curve of the clinical value of *PRR34*‐*AS1* abnormal methylation in AML. (A) All AML patients; (B) non‐APL‐AML patients; AUC, area under the ROC curve; non‐APL, non‐acute promyelocytic leukemia; (C–E) effect of *PRR34*‐*AS1* methylation on OS in AML. Patients were classified into *PRR34*‐*AS1* hypomethylated and hypermethylated groups based on ROC curve analysis. (C) whole‐cohort AML patients. (D) non‐APL‐AML patients. (E) cytogenetically normal AML (CN‐AML) patients. (F) Effect of *PRR34*‐*AS1* methylation on OS using TCGA database. One hundred and ninety‐four AML patients were grouped into hypomethylated and hypermethylated groups based on the median level of *PRR34*‐*AS1* methylation, and survival analysis was conducted

The ROC curve was plotted to evaluate the diagnostic value of *PRR34*‐*AS1* methylation in AML. The results showed that the *PRR34*‐*AS1* methylation level may be a potential marker for distinguishing AML (especially non‐APL‐AML) patients from normal controls (95% CI = 0.513–0.722, *p* = 0.060, AUC = 0.617; 95% CI = 0.529–0.749, *p* = 0.032, AUC = 0.639; Figure 3A,B). Patients were then divided into *PRR34*‐*AS1* hypermethylated group and hypomethylated group based on ROC analysis in order to explore the relationship between *PRR34*‐*AS1* methylation and different clinical parameters in AML. Analysis showed no significant differences between the levels of methylation and gender, age, hemoglobin, platelets, and BM blasts between the two groups (*p* > 0.05; Table 5). Similarly, *PRR34*‐*AS1* methylation showed no significant correlation with eight genetic mutations (*p* > 0.05; Table 5). However, patients with hypomethylated *PRR34*‐*AS1* had a higher WBC count (*p* = 0.006) and showed a low frequency of favorable karyotypes compared with hypomethylated group [28% (7/25), *p* = 0.071; Table 5].

**TABLE 5:** Comparison of clinical characteristics between *PRR34*‐*AS1* hypomethylated and *PRR34*‐*AS1* hypermethylated group

Patient's parameters	Hypermethylated (*n* = 49)	Hypomethylated (*n* = 35)	*p* value
Sex, male/female	24/25	20/15	0.511
Median hemoglobin, g/L (range)	76 (32–138)	78 (42–135)	0.969
Median age, years (range)	52 (18–83)	57 (20–85)	0.162
Median WBC, ×10^9^/L (range)	9.4 (0.3–107.0)	35.6 (0.9–528)	0.006
Median platelets, ×10^9^/L (range)	34 (5–234)	52 (9–264)	0.211
BM blasts, % (range)	50 (1–94)	35 (5.5–99.0)	0.485
FAB subtypes			0.621
M0	0 (0%)	1 (2.9%)	
M1	4 (8.2%)	2 (5.7%)	
M2	15 (30.6)	15 (42.9%)	
M3	14 (28.6%)	6 (17.1%)	
M4	11 (22.4%)	6 (17.1%)	
M5	3 (6.1%)	4 (11.4%)	
M6	2 (4.1%)	1 (2.9%)	
Karyotype classification			0.071
Favorable	18 (36.7%)	7 (20%)	
Intermediate	18 (36.7%)	23 (65.7%)	
Poor	10 (20.4%)	4 (11.4%)	
No data	3 (6.1%)	1 (2.9%)	
Karyotype			0.065
Normal	15 (30.6%)	16 (45.7%)	
t (8;21)	5 (10.2%)	1 (2.9%)	
t (15;17)	13 (26.5%)	6 (17.1%)	
t (9;22)	0 (0%)	2 (5.7%)	
11q23	1 (2%)	0 (0%)	
−5/5q−	1 (2%)	0 (0%)	
−7/7q−	3 (6.1%)	1 (2.9%)	
complex	2 (2.4%)	6 (17.1%)	
No data	9 (18.4%)	0 (0%)	
Gene mutation			
*CEBPA* (+/−)	6/38	3/27	0.731
*NPM1* (+/−)	2/42	2/28	>0.999
*FLT3*‐*ITD* (+/−)	3/41	2/28	>0.999
*C*‐*KIT* (+/−)	2/42	1/29	>0.999
*N*/*K*‐*RAS* (+/−)	3/41	2/28	>0.999
*IDH1*/*2* (+/−)	5/44	2/28	0.161
*DNMT3A* (+/−)	2/37	1/24	>0.999
*U2AF1* (+/−)	1/38	2/24	0.562
CR (+/−)	18/21	9/17	0.154

Abbreviations: BM, bone marrow; CR, complete remission; FAB, French–American–British; WBC, white blood cells.

### 3.6 Association between *PRR34*‐*AS1* methylation and clinical outcomes in AML patients

Correlation analysis was performed between *PRR34*‐*AS1* methylation and clinical outcomes to explore the value of *PRR34*‐*AS1* methylation in the prognosis of AML patients. Analysis methylation levels were not significantly correlated with CR of AML patients. Interestingly, OS of patients with hypomethylated *PRR34*‐*AS1* was shorter than that of patients with hypermethylated *PRR34*‐*AS1* in the whole‐cohort AML and non‐APL‐AML (*p* = 0.010, Figure 3C; *p* = 0.032; Figure 3D). Similar results were obtained through the analysis of the TCGA data sets (*p* < 0.001; Figure 3F). Moreover, Cox proportional hazards model supported that the hypomethylation of *PRR34*‐*AS1* was an independent risk factor for OS among whole‐AML and non‐APL‐AML patients (Table 6).

**TABLE 6:** Univariate and multivariate analyses of prognostic factors for overall survival in whole‐cohort‐AML and non‐APL patients

Variables	Whole‐cohort‐AML (*n* = 84)				Non‐APL‐AML (*n* = 64)			
	Univariate analysis		Multivariate analysis		Univariate analysis		Multivariate analysis	
	HR (95% CI)	*p* value	HR (95% CI)	*p* value	HR (95% CI)	*p* value	HR (95% CI)	*p* value
WBC	1.895 (1.124–3.195)	0.016	0.856 (0.443–1.652)	0.643	1.443 (0.835–2.493)	0.189	0.691 (0.341–1.402)	0.306
Age	2.632 (1.550–4.470)	<0.001	1.846 (1.076–2.297)	0.031	1.814 (1.042–3.158)	0.035	1.370 (0.754–2.486)	0.301
*PRR34‐AS1* methylation	0.522 (0.310–0.879)	0.014	0.578 (0.329–1.017)	0.057	0.573 (0.331–0.994)	0.047	0.483 (0.264–0.883)	0.018
Karyotype risk	1.754 (1.263–2.436)	0.001	1.572 (1.076–2.297)	0.019	1.496 (0.994–2.251)	0.054	1.610 (1.009–2.570)	0.046
*CEBPA* mutation	2.137 (0.896–5.096)	0.087	1.828 (0.750–4.458)	0.185	1.949 (0.811–4.685)	0.136	2.139 (0.878–5.212)	0.094
*NPM1* mutation	1.453 (0.523–4.041)	0.474	—	—	1.190 (0.422–3.354)	0.743	—	—
*FLT3*‐ITD mutation	0.712 (0.256–1.980)	0.515	—	—	0.833 (0.296–2.348)	0.730	—	—
*c*‐*KIT* mutation	0.583 (0.142–2.405)	0.456	—	—	0.388 (0.053–2.823)	0.349	—	—
*N*/*K*‐*RAS* mutation	1.128 (0.403–3.152)	0.819	—	—	0.918 (0.324–2.601)	0.871		
*DNMT3A* mutation	0.964 (0.346–2.682)	0.944	—	—	0.770 (0.274–2.168)	0.621	—	—

Note

Variables including age (≤60 vs. <60 years), WBC (≥30×10^9^ vs.<30×10^9^/L), *PRR34*‐*AS1* methylation (unmethylated vs. methylated), karyotype risk (favorable vs. intermediate vs. poor), and gene mutations (mutant vs. wild type).

Multivariate analysis includes variables with *p* < 0.200 in univariate analysis.

Abbreviations: AML, acute myeloid leukemia; CI, confidence interval; CN‐AML, cytogenetically normal AML; HR, hazard ratio; non‐APL‐AML, non‐acute promyelocytic leukemia‐AML; WBC, white blood cells.

## 4 DISCUSSION

AML is a complex disease with high heterogeneity at the molecular level and in clinical symptoms.^40^ Most of the previous studies largely focused on protein‐coding genes as key components of disease progression. A few studies have explored the role of non‐coding genes in the progression of AML. Studies report that lncRNAs have a diagnostic value and prognostic potential in several types of cancer, including AML.^41^

The present study explored the correlation between *PRR34*‐*AS1* expression and prognosis of AML. Kaplan–Meier analysis showed that OS in AML patients with higher *PRR34*‐*AS1* transcript level was significantly shorter compared with that of patients with low expression levels. Notably, high heterogeneity of AML may interfere with effective diagnosis, prognosis, and identification of predictive biomarkers.^42^ Analysis of GEO and TCGA data sets showed a significant increase in the expression of *PRR34*‐*AS1* in BM specimens of AML patients. In addition, patients with high expression of *PRR34*‐*AS1* had a significantly shorter OS than the low expression group. However, Cox analysis showed that *PRR34*‐*AS1* expression was not an independent factor affecting OS in AML patients. This finding implies that multiple molecular mechanisms may contribute to the differential expression of *PRR34*‐*AS1*, and *PRR34*‐*AS1* may be involved in the early stages of AML disease progression. A previous study by Kang et al. used array comparative genomic hybridization to explore copy number variation (CNV) in *PRR34*‐*AS1*. The findings for the study showed that an increased copy number of *PRR34*‐*AS1* was correlated with early recurrence and poor DFS in cholangiocarcinoma patients.^27^ Findings of the present study showed that high expression of *PRR34*‐*AS1* was associated with a reduced CR rate. Multivariate analysis further showed a significant correlation between the high expression of *PRR34*‐*AS1* and low CR in AML patients. This suggested that high *PRR34*‐*AS1* expression may be one of the related factors contributing to the poor efficacy of chemotherapy in AML patients. These findings demonstrated that high *PRR34*‐*AS1* expression may be associated with poor chemotherapeutic efficacy and poor prognosis in AML patients. Notably, minimal residual disease (MRD) monitoring helps evaluate the efficacy of induction therapy and for monitoring the early recurrence of AML, to allow the adjustment of treatment strategies. However, there were fewer patients with serial samples in this study, the role of *PRR34*‐*AS1* expression in MRD monitoring and recurrence of AML was not explored. Further studies with a longer follow‐up and a bigger sample size should explore the role of *PRR34*‐*AS1* expression in MRD monitoring and recurrence of AML.

Previous studies report that epigenetic disorders play a vital role in the pathogenesis of AML. DNA methylation can be used as an epigenetic modification to regulate gene expression.^43^ In this study, MSP and BSP were used to detect and verify the levels of methylation in the DMR of *PRR34*‐*AS1*. The relationship between the methylation levels of *PRR34*‐*AS1* and the expression of this gene was also explored. Analysis showed that the DMR of *PRR34*‐*AS1* displayed a pattern of hypomethylation, compared with the controls; however, there was no significant difference between the two groups. Spearman correlation analysis showed that *PRR34*‐*AS1* hypomethylation was associated with its expression. This finding implies that hypomethylated DMR of *PRR34*‐*AS1* may be an important regulatory mechanism for the expression of *PRR34*‐*AS1* in AML. The effect of the abnormal methylation of *PRR34*‐*AS1* on the prognosis of AML was explored. The findings showed that the hypomethylation of *PRR34*‐*AS1* was correlated with a shorter OS of AML patients. Furthermore, multivariate analysis verified that *PRR34*‐*AS1* hypomethylation was an independent risk factor for OS. However, the small sample size used in the study may have resulted in relative errors in the results. These results should, therefore, be verified using larger sample sizes. Similar results were obtained from analysis using DiseaseMeth version 2.0 and TCGA database. The findings showed a significant decrease in the methylation level of *PRR34*‐*AS1* promoter in AML and the short OS for patients with hypomethylated *PRR34*‐*AS1* compared with hypermethylated patients.

Two major hypomethylating agents (HMAs), decitabine and azacytidine, have been used clinically for the treatment of elderly AML patients not suitable for or decline intensive remission therapy. However, primary or secondary failure occurs in about 80% of treated patients.^44^ Although reactivated tumor suppressor genes have been supposed as the major antileukemic mechanism of HMAs, there is a concern that specific oncogenes will also be reactivated by demethylation.^45^ Preliminary findings of the current study show that *PRR34*‐*AS1* may be an oncogenic lncRNAs. However, the exact role of *PRR34*‐*AS1* in leukemogenesis should be explored further. Moreover, further studies should explore whether *PRR34*‐*AS1* can be reactivated after treatment with HMA and the impact of its reactivation.

Although the present study uncovered some insightful findings, it had a number of shortcomings. First, the clinical sample size was small included patients with normal karyotypes and related gene mutations. Second, RQ‐PCR and other detection methods used in the study are less accurate than high‐throughput sequencing. Additionally, the experimental results were not verified through cell function experiments. Moreover, this was a preliminary study on the relationship between *PRR34*‐*AS1* and AML. Therefore, more studies should be conducted to verify the results and uncover the underlying mechanisms of *PRR34*‐*AS1*.

In summary, the findings of this study show that high *PRR34*‐*AS1* expression may be associated with poor chemotherapeutic efficacy and poor prognosis in AML patients. In addition, higher expression of *PRR34*‐*AS1* was associated with the hypomethylation of its promoter, and hypomethylation of *PRR34*‐*AS1* may affect the prognosis of AML patients.

## DATA AVAILABILITY STATEMENT

The data sets used and/or analyzed during the current study are available from the corresponding author on reasonable request.

## CONFLICT OF INTERESTS

The authors declare that they have no competing interests.

## AUTHOR CONTRIBUTIONS

J Q and F‐y N conceived and designed the experiments; F‐y N and YG performed the experiments; F‐y N, G‐k S, and Z‐j X analyzed the data; Z‐j X, J‐d Z, T‐j Z, and J‐y L collected the clinical data; J L, J‐c M, and J Q offered technique and language support; F‐y N wrote the paper. All authors read and approved the final manuscript.

## ETHICS APPROVAL AND CONSENT TO PARTICIPATE

The study was approved by the Clinical Research Ethics Committee of the Affiliated People's Hospital of Jiangsu University.

## CONSENT FOR PUBLICATION

Written informed consent was obtained from all enrolled individuals before their participation.

## Supporting information

Table S1
