# Supplementary material for: Abnormal expression and methylation of PRR34‐AS1 are associated with adverse outcomes in acute myeloid leukemia
Source: Cancer Med. 2021 Jul 5;10(15):5283–96. doi: 10.1002/cam4.4085 (PMC8335806; doi:10.1002/cam4.4085)
Supplement: Supplementary file 1 — Table S1 [file CAM4-10-5283-s001.docx]

Supplementary Table 1. Combined chemotherapy protocols for AML patients in this study

| Population | Induction therapy | consolidation treatment after CR | maintenance therapy |
| --- | --- | --- | --- |
| Non-APL patients | 1-2 cycle of daunorubicin (45 mg/m^2^ daily for 3 days) or Idarubicin (10-12 mg/m^2^ daily for 3 days) in combination with cytarabine (100 mg/m^2^ daily for 7 days). | high-dose cytarabine (3 g/m^2^ on days 1, 3, 5); or   1. 2 cycles of mitoxantrone (8 mg/m^2^ for 3 days) in combination with cytarabine (100 mg/m^2^ daily for 7 days); 2. 1-2 cycles of homoharringtonine (2 mg/m^2^ daily for 7 days) combined with cytarabine (100 mg/m^2^ daily for 7 days); 3. 1-2 cycles of etoposide (100 mg/m^2^ daily for 3 days) in combination with cytarabine (100 mg/m^2^ daily for 7 days). |  |
| APL patients | 45mg/m^2^ oral all-trans retinoic acid (ATRA) per day, 45mg/m^2^ intravenous daunorubicin for 3 days and 100 mg/m^2^ cytarabine for 7 days. | 1. daunorubicin (45 mg/m^2^ for 3 days) and cytarabine (100 mg/m^2^ for 7 days)； 2. mitoxantrone (8 mg/m^2^ per day for 3 days) and cytarabine (100 mg/m^2^ for 7 days); 3. homoharringtonine (2 mg/m^2^ daily for 7 days) and cytarabine (100 mg/m^2^ for 7 days). | Oral mercaptopurine (50 mg/m^2^ per day), oral methotrexate (15 mg/m^2^ per week), and oral ATRA (45 mg/m^2^ per day for 15 days every 3 months) over 2 years |
| AML patients (age >65) | cytarabine (10 mg/m^2^ q12 hr for 14 days), homoharringtonine (1 mg daily for 14 days), and granulocyte-colony stimulating factor (G-CSF) (200μg/m^2^ for 14 days). | 1. 2 cycle of cytarabine (100 mg/m^2^ daily for 7 days) combined with 8 mg/m^2^ mitoxantrone (8 mg/m^2^ for 3 days);   (b) 1-2 cycles of cytarabine (1000 mg/m^2^ on days 1, 3, 5, and 7). |  |
